# A Systematic Review of Community Readiness Tool Applications: Implications for Reporting

**DOI:** 10.3390/ijerph120403453

**Published:** 2015-03-24

**Authors:** Iordan Kostadinov, Mark Daniel, Linda Stanley, Agustina Gancia, Margaret Cargo

**Affiliations:** 1 School of Population Health, University of South Australia, Adelaide, SA 5001, Australia; E-Mails: Iordan.Kostadinov@mymail.unisa.edu.au (I.K.); Mark.Daniel@unisa.edu.au (M.D.); Agustina.Gancia@mymail.unisa.edu.au (A.G.); 2 Department of Medicine, St. Vincent’s Hospital, The University of Melbourne, Fitzroy, VIC 3065, Australia; 3 South Australian Health and Medical Research Institute, Adelaide, SA 5001, Australia; 4 College of Natural Sciences, Tri-Ethnic Center, Colorado State University, Fort Collins, CO 80523, USA; E-Mail: Linda.Stanley@colostate.edu

**Keywords:** community readiness, evaluation, community interventions, systematic review

## Abstract

Background: A systematic review characterised and synthesised applications of the Community Readiness Tool (CRT) and synthesised quantitative results for readiness applications at multiple time points. Methods: Eleven databases in OvidSP and EBSCHOhost were searched to retrieve CRT applications. Information from primary studies was extracted independently by two researchers. Results: Forty applications of the CRT met inclusion criteria focussing on 14 different health and social issues. The community of interest was most often defined solely on the basis of its geographical location (52.5%). Most studies used the CRT to plan (85%) and/or evaluate programs (40%). The CRT protocol was modified in 40% of studies. Six applications evaluated readiness at multiple time points, however limited reporting in primary studies precluded any synthesis of results. Applications identified methodological rigour, contextual information and community engagement as strengths, and time and resource costs as limitations. Conclusions: The CRT is well suited for planning and evaluating complex community health interventions given its flexibility to accommodate diverse definitions of community and issues. CRT applications would benefit from improved reporting; reporting recommendations for use of the CRT are outlined.

## 1. Introduction

Complex community interventions are often referred to as “*context-dependent*” to acknowledge the important influence of local contextual conditions in shaping public health intervention outcomes. Context-dependency most often reflects the definition of community as locality, expressed by its geographical boundaries (*i.e.*, suburb, town, city, metropolitan area). Communities, however, are also defined by people or social entities sharing a common interest, culture, values, norms or characteristics [1,2,3]. These definitions of community are not mutually exclusive. It is not uncommon for public health intervention efforts to be defined by people sharing a common interest within a geographically defined area.

Assessing the heterogeneity of local conditions in relation to the different expressions of “*community*” is a challenge in the planning and evaluation of public health intervention programs. Community and local environments are characterised by a range of factors including both objective and subjective aspects of social and built environments. Salient features of the environment can range from demographics (such as age and gender) and socioeconomic indicators (education, occupation and income levels) to public open space, community resources, and broader community readiness to change. Although objective measures may be obtained from administrative sources such as census data, important subjective community-level factors relevant to health interventions can be difficult to capture.

Community readiness to mobilise around a health issue has been identified as an important contextual factor to account for in the planning and evaluation of complex interventions [4]. A community’s readiness to mobilise can impact on program success [4]. For example, low levels of readiness may result in intervention staff facing significant challenges [5] in mounting an intervention due to inadequacies in the level of local support, leadership or resources. By contrast, implementation may be facilitated in communities with a high level of readiness owing to the combination of leadership, presence of in-kind or financial resources and local knowledge or expertise. There is little evidence on the specific intervention strategies needed to increase community readiness over time, the timeframes required for communities to mobilise, and whether the timeframes vary for different types of issues or communities.

Quantifying community readiness is complex, and several tools have been developed for this purpose [4,6,7,8,9]. One widely used and flexible tool for measuring community readiness has been developed by Edwards and colleagues at Colorado State University [4,10]. This community readiness tool (CRT) was originally developed to understand the types of drug and alcohol abuse prevention programs which were best suited to small communities in the USA. The CRT is based on the community readiness model (CRM), initially underpinned by the personal stages of change model [11] and community development principles [12]. The CRM built upon these principles and expanded on them to include new dimensions which were unique to communities and program development as well as introducing stages within each dimension to track the progress of a community from a state of no awareness to the community taking full ownership of an issue. The CRM was refined through expert consultation and the application of psychometric principles. In addition, a protocol to measure community readiness (the CRT) was defined and applied. The CRM defines the following six dimensions that are scored for readiness through the CRT: Community Efforts, Community Knowledge of the Efforts, Leadership, Community Climate, Community Knowledge about the Issue and Resources Related to the Issue [13].

*A priori* definitions of the issue and the target community are the starting point for applying the CRT, as readiness is issue- and community-specific. A set of 20 core and 16 optional interview questions is then adjusted for the specific issue and community. Semi-structured interviews of approximately 45–60 min are conducted individually with four to six key stakeholders in each community. Interviews are transcribed, and each dimension is scored on a nine-point anchored rating scale using two scorers for each interview. The six dimension scores are then averaged to give each community an overall community readiness score with a range of one to nine, with one denoting no awareness, and nine denoting high levels of community ownership.

The community focus of the CRM and CRT is highly relevant to the planning and evaluation of public health intervention programs. Since its introduction to the field in 1997, the CRT has evolved with protocol improvements to the scoring and interview scripts [14] and applications for a wide variety of health and social issues. Despite its growing popularity, however, little is known about the scope of its application. That is, whether it is most often applied to assess the readiness of single or multiple communities and whether its scope of use extends to the evaluation of large-scale population health interventions focused on community-level change. In addition, whilst there is a standard protocol for applying the tool, the extent of modifications required to tailor question wording to the relevant issue, and the reasons for these modifications, remain unclear.

To understand the scope of use of the CRT and the CRM upon which it is based, a systematic review of all applications published in the academic literature was conducted. The specific research objectives were to:

(1) Characterise the types of communities, issues and impetus of use for the CRT and CRM in published literature;
(2) Identify the perceived strengths and weaknesses of the CRT and CRM based on study authors’ experiences;
(3) Assess the extent to which the CRT and CRM have been modified in the empirical literature and to explore the reasons for these modifications;
(4) Describe how community readiness results are reported in the empirical literature; and
(5) Synthesise readiness results across evaluation applications of the CRT and CRM across multiple time points.

## 2. Methods

Any application of the CRT or CRM published in peer reviewed journals was eligible for inclusion; papers that did not apply the CRT and report on findings were excluded. Searches were conducted on 7 August 2013 using the OvidSP (Medline, Embase and ICONDA) and EBSCOhost (ERIC, Psychinfo, CINAHL, PsycARTICLES, PsycBOOKS, PsycCRITIQUES, PsycEXTRA, PsycINFO) search platforms. The search term “*community readiness*” was used in both search platforms; all articles from 1997 onwards were considered, without further restrictions applied.

A data extraction tool was developed and pre-tested by the first and last authors on three community readiness articles meeting the inclusion criteria. The tool was designed to capture information on quantitative variables and qualitative information pertaining to review objectives. The extraction tool was modified according to the pilot-testing and used in the final extraction.

Data extraction was completed independently by two researchers. One researcher (IK) had previously applied the CRT and had a high level of expertise with both the CRT and CRM; the second researcher (AG) reviewed the CRT Training Manual and additional training materials prepared by the Tri-Ethnic Centre for Prevention Research.

Quantitative variables extracted from each article included: year of publication, country, location (urban or rural), reported modification of the tool (yes/no), number of communities, number of interviews per community, consensus scores (dimension-specific and overall scores) (yes/no), standard deviations (dimension-specific and overall scores) (yes/no), and reporting format of community readiness scores and the standard deviations. Variables had dichotomous, continuous or nominal response categories.

Descriptive qualitative information extracted from each article pertained to: readiness issue, types of key informants used, definition of community, impetus for usage of the CRT, reason for modification, modification description, perceived strengths and weakness of the CRT, perceived strengths and weaknesses of the CRM, and format for reporting of results. Categorical responses were inductively generated based on the responses provided in the original studies.

### Analysis

Descriptive statistics (frequencies, means, and standard deviation) were computed using Microsoft Excel 2010 software. Open-ended responses for qualitative questions were content analysed for nominal level categories. Discrepancies in categorisation were reconciled through discussion between the two data extractors.

## 3. Results

**Figure 1:**
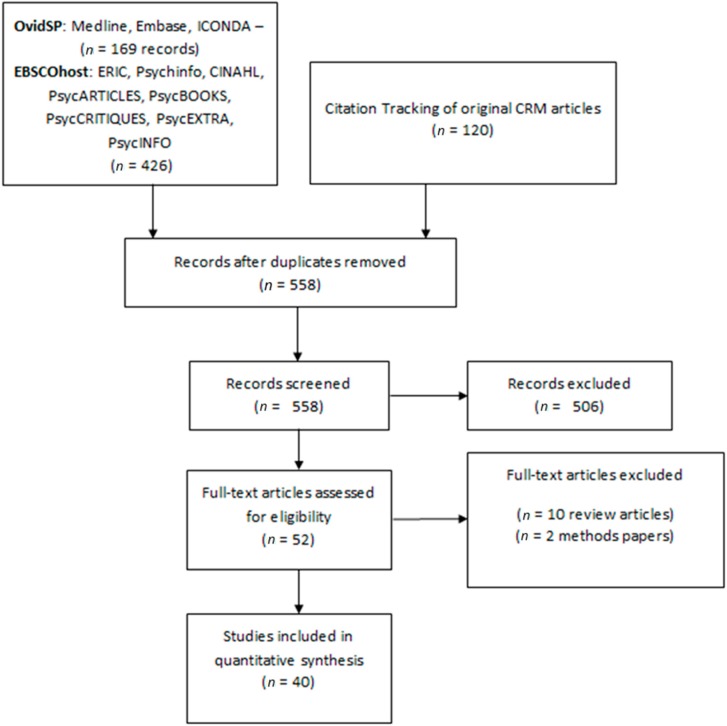
PRISMA flow diagram describing the selection of studies applying the Community Readiness Tool.

The final sample contained 40 unique studies published between 1999 and 2013. Databases searched through OvidSP returned 169 records, whilst databases searched through EBSCOhost returned 426 records. A further 120 studies were identified by tracking the citations of the original published studies of the CRT and CRM. One-hundred ninety-eight duplicate studies were removed leaving 558 unique records for screening; 506 records were excluded based on the title and abstract not referring to either the CRT or the CRM. The full text was retrieved for 52 studies; 10 papers were excluded for not applying the tool and two papers excluded for only describing proposed methods rather than reporting on an application of the CRT. This process is illustrated in the PRISMA diagram (Figure 1).

The application publication rate increased from 1.5 studies per year between 1999 and 2006 to 4.4 studies per year from 2007 onwards. Studies were conducted predominantly in the USA (85%) (Table 1). Primary studies applied the CRT either in urban (30%), rural (47.5%) or both settings (17.5%). The rurality of the community was unclear in two of the applications (5%). Definitions of community varied, but most commonly were based solely on their geographical boundaries; e.g., rural towns (22.5%), counties/administrative units (22.5%), and urban cities (7.5%). Other types of communities which were bound by shared interests (7.5%) or ethnic/indigenous identity (22.5%) and organisations (15%) were also common. One application had no clear definition of community (2.5%).It should be noted that although these types of communities were not solely geographical, all of them had a geographical component (e.g., Hispanic residents in Nashville, Yup’ik youth in a village, residents of public housing developments in Boston). The number of communities in each study varied between one and 102 (mean = 11.7, SD = 19.2; median = 4); however, 90% of studies had less than 22 communities, 60% had less than 10 communities and 30% of studies focussed on one community. The number of interviews per community varied between one and 33 (mean = 7.3, SD = 5.9; median = 6), with 15% of studies using less than the recommended four interviews per community. The number of interviews was unclear in six studies.

As shown in Table 1, the CRT was applied to a range of issues, the most prominent of which were alcohol/drug related issues (17.5%) and tobacco control (17.5%). Childhood obesity (12.5%), HIV/AIDS (10%), cancer (10%), disability and trauma (7.5%), and domestic violence (7.5%) were also issues around which communities mobilised. Bicycle helmet use, Native American cultural programs, cardiovascular disease, youth violence, Gay, lesbian, bisexual and transgender (GLBT) services, general health, and multiple issues were addressed as single issues in the remaining 17.5% of studies.

**Table 1:** Characteristics of published studies included in this systematic review (*n* = 40).

Characteristic	*n*	%	Characteristic	*n*	%
**Nation of Community**			**Readiness Issue**		
USA	33	82.5	Alcohol and drug related	7	17.5
Australia	2	5	Tobacco control	7	17.5
Bangladesh	1	2.5	Childhood obesity	5	12.5
Canada	1	2.5	HIV/AIDS	4	10
India	1	2.5	Cancer	4	10
Liberia	1	2.5	Disability and trauma	3	7.5
Unclear	1	2.5	Domestic issue	3	7.5
--	--	--	Bicycle helmet use	1	2.5
**Urban/Rural**	--	--	Native American cultural programs	1	2.5
Urban only	12	30	Cardiovascular disease	1	2.5
Rural only	19	47.5	Youth violence	1	2.5
Both urban and rural	7	17.5	Services to GLBT	1	2.5
Unclear	2	5	General Health	1	2.5
--	--	--	Multiple issues	1	2.5
**Definition of community**	--	--	--	--	--
*Geographic*	*21*	*52.5*	**Modification**	--	--
Country Town	9	22.5	Modifications to the methodology	16	40
County/Administrative unit	9	22.5	No changes to the methodology	24	60
Urban City	3	7.5	--	--	--
*Non-Geographic*	*18*	*45*	--	--	--
Ethnic/Indigenous group	9	22.5	**Reason for Modification (*n* = 16)**	--	--
Organisation	6	15	Better fit local context	10	62
Shared interest	3	7.5	Reduce time/effort of administration	2	13
*No clear definition*	*1*	*2.5*	Fit available data	1	6
--	--	--	No explicit reason	3	19
**Number of communities**	--	--	**Reporting CR scores**	--	--
1	12	30	Both overall and dimension scores	17	42.5
2–9	11	27.5	Only overall score reported:	19	47.5
10–22	11	27.5	No CR scores reported	4	10
24–102	7	12.5	--	--	--
unclear	1	2.5	--	--	--
**Number of reasons for use of CRT**					
1	27	67.5	--	--	--
2	13	32.5	--	--	--
	--	--	--	--	--
Reasons behind usage ^*^	--	--	**Reporting of Standard Deviation**	--	--
Planning prevention efforts	34	85	Both overall and dimension SD	1	2.5
Program evaluation	16	40	Only overall SD	3	7.5
Community engagement	1	2.5	No SD reported	36	90
Improving CR methodology	1	2.5	--	--	--
To select intervention communities	1	2.5	--	--	--

^*^ Because more than one response can be given, the total frequency and percentages exceed the number of papers.

Many studies (42.5%) reported both overall and dimension community readiness scores, with 47.5% reporting only the overall community readiness scores. Four studies (10%) did not report any community readiness scores. Of these studies, one reported only changes in community readiness over time without stating overall scores [15], one reported only changes in scores between versions of the tool without stating overall scores [16], and two discussed using the CRT without reporting any scores [17,18]. The majority of studies did not report standard deviations (90%).

Of six studies applying the CRT at multiple time points [5,15,19,20,21,22], one study reported mean scores and standard deviations [5], three studies graphed the results [15,19,20], one study provided readiness stage names only (*i.e.*, baseline = “*denial*” and follow up = “*vague awareness*”) [21], and one study reported mean scores without standard deviations [22]. The changes in community readiness scores within these studies ranged from an increase of 0.5 to 5 points. The timeframe between baseline and follow up assessments ranged from one to three years. The study with the lowest gains in readiness scores (changes from readiness level 2 to readiness level 3) involved the least intense intervention: small community grants (less than USD$2000) given to two communities over two years to run education sessions around traumatic brain injuries [21]. Jason *et al.* [15] reported the correlation between years voluntarily spent in intervention and increased readiness scores: each year of sustained intervention correlated with a mean increase of 0.6 on the community readiness score [15].Pre-experimental (*n* = 3), quasi-experimental (*n* = 2), and experimental (*n* = 1) designs were used to evaluate interventions in a community (*n* = 2) or multiple communities (*n* = 4). Community readiness was modelled descriptively as an outcome variable in all studies. Only one study assessed the relationship between community readiness and a health outcome: Millar *et al.* [19] reported an inverse relationship between community readiness and the prevalence of childhood obesity. Other studies reported separate descriptive analyses for health outcomes and community readiness where improvements in health outcomes corresponded to greater community readiness scores at baseline. Given the lack of consistent reporting of dimension and overall scores, and the absence of standard deviations, it was not possible to quantitatively synthesise the results across studies.

Some studies (32.5%) reported more than one reason for using the CRT. Planning future prevention efforts was the most common reason (85%). These studies used the community readiness score to tailor interventions to the local context. In 40% of applications, the CRT was used to assess current levels of readiness for evaluation purposes or to match intervention and comparison communities prior to intervention. Six studies (15%) used the CRT both pre- and post-intervention to measure program success. Other reasons behind the usage of the CRT included engagement of the community prior to any intervention efforts (2.5%), improving community readiness methodology and scoring rigour (2.5%), and selecting communities where the intervention was most likely to succeed (2.5%).

The CRT protocol allows for minor modifications to the methodology and interview scripts to tailor the tool to the particular issue and community at hand [13]. However, many of the studies (40%) reported substantial changes to the application of the CRT beyond these usual minor adjustments. In six studies (15%), substantial changes were made to the core questions with either removal or addition of questions. Six other studies (15%) made more significant changes, including changing dimensions, adding new dimensions, or altering existing dimensions to better fit the subject area. Eight other studies (20%) changed the data collection method from the traditional one-on-one interview to a group interview, online interview, or obtaining data from other, non-interview, sources. Two (5%) studies changed the scoring procedures and scales, using their own scales (one was changed to a score between 0 and 1 [23], the other to a score out of 4 [24]) instead of the usual 9 point anchored rating scale. Some studies made multiple changes to the CRT protocol.

Of the sixteen studies that made significant modifications to the tool, ten aimed to improve the fit of the tool with the local context (62%). Two studies (13%) altered the CRT to reduce the time and effort required for administration, and one study (6%), not having completed a regular community readiness assessment, made changes to fit the CRT around the available data. Three studies (19%) gave no explicit reason to support the modification.

Few studies identified limitations to the CRM or CRT (Table 2). With respect to the CRM, only 10% discussed limitations. These studies commented that the CRM was not comprehensive enough, with economic and social factors not clearly reflected in the model and dimensions perceived as narrowly defined (7.5%). One study (2.5%) pointed to a perceived lack of rigour in the development of the tool and its dependency on key informant perspectives, and recommended further validation of the model [25]. Limitations of the CRT were reported in 30% of studies. The most commonly noted limitation was the substantial time and resource commitment necessary to complete the assessment (12.5%). The issue of subjective scoring, in which qualitative interview results are scored by researchers on an anchored rating scale rather than captured through the use of an objective standard, arose in some studies (10%). Other limitations included: response bias by the key informants; the ever changing and transient nature of readiness not being suited to measurement at a single point in time; and limited power to statistically detect significant findings given relatively few interviews per community and few communities.

Most studies explicitly discussed the strengths of the CRM (65%), with some discussing more than one strength. Many (25%) praised the CRM for its ability to provide intervention strategies tailored to the community’s level of readiness, whilst others (20%) found it provided key contextual information which improved intervention development or evaluation. A few studies highlighted its theory-based framework (10%), commended it for its adaptability to different issues and communities (7.5%), and found that it contributed to the community development agenda by identifying and engaging key stakeholders within the community (10%).

**Table 2:** Limitations and strengths of the community readiness model (CRM) and community readiness tool (CRT) as discussed by the studies included in this review (*n* = 40).

Characteristic	*n*	%
**Limitations of CRM**		
Not comprehensive enough	3	7.5
Development and reliance on key informants	1	2.5
None discussed	36	90
**Limitations of CRT**		
High time and resource commitment in administration	5	12.5
Subjective scoring	4	10
Transient nature of readiness	1	2.5
Statistical power issues	1	2.5
Key informant bias	1	2.5
None discussed	28	70
^*^ Strengths of the CRM		
Provides tailored intervention strategies	10	25
Provides key contextual information	8	20
Theory based framework	4	10
Adaptive	3	7.5
Contributes to community development	4	10
None discussed	14	35
^*^ Strengths of the CRT		
Perceived methodological rigor	10	25
Built relationships/good starting point for intervention staff	6	15
Assessment of community prior to intervention	5	12.5
Strong qualitative data collected	3	7.5
Adaptive	2	5
Community ownership of tool	1	2.5
Easy scoring	1	2.5
Lack of outside experts needed	1	2.5
None Discussed	22	55

^*^ Because more than one response can be given, the total frequency and percentages exceed the number of papers.

Twenty-five percent of studies favourably discussed perceived methodological rigour of the CRT (*i.e.*, sampling of diverse community members, use of multiple interviews, multiple scorers for each interview), with others (5%) praising its ability to adapt to the issue at hand. The lack of reliance on outside experts (2.5%), community ownership of the program (2.5%), and easy scoring procedure (2.5%) also were identified as assets of the CRT. The qualitative data gathered by the tool was seen as a strength in three of the studies. Some studies commended the CRT for its assessment of community support prior to prevention programs (12.5%), and another 15% found that the CRT helped build relationships and was a useful starting point for intervention staff. A full set of results for all studies are provided in Tables S1 and S2 in the supplementary material.

### 3.1. Discussion

This is the first time a systematic review of the CRT applications has been conducted. A systematic search for published applications of the CRT identified 40 relevant studies. The majority of studies were based in the USA where the CRT was first developed. The versatility and flexibility of the CRT is exemplified in its application to a diverse range of health and social issues and definitions of community. The review was unable to either quantitatively synthesise results across evaluation studies which assessed readiness at multiple time points, due to reporting limitations and considerable modifications in application of the tool. Key findings are highlighted below with implications culminating in a preliminary set of recommendations to improve reporting in future studies.

The primary studies included in the review mirror the World Health Organisation definitions of community [1]. Although applications of the CRT tended to emphasise geographically bounded communities (*i.e.*, cities, towns or administrative areas), organisational communities, such as health centres, care facilities or schools were also featured. It is of interest to note that many of the ethnic and Indigenous communities that mobilised around a particular issue were also geographically and social-network bound, as reflected in Latino women mobilising within cities [26], Korean communities mobilising within San Francisco [27], the Indigenous Yup’ik community mobilising within a small village [20], or the Native American community mobilising within the state of Wisconsin [14]. It was less common to find applications of non-geographic communities brought together by a shared interest. Where shared interests were utilised, they included sexual orientation [28], cycling [29], or use of a community health centre [30].

As illustrated in Table 1, the CRT was applied to a broad range of issues highly relevant to public health. The flexibility of the tool in accommodating a broad range of communities and issues sees it well-suited to the participatory planning and evaluation of complex community health promotion interventions. In these interventions salient health and social issues emerge from researchers and practitioners working with communities which, themselves, may crystallise through the participatory process. Thus, the CRT aligns with a “*best process*” approach to program planning and evaluation supported by such time-honoured models as Precede-Proceed [3] as well as more recent developmental evaluation approaches [31]. Involving the community in the planning and evaluation of community health promotion programs is a longstanding health promotion principle. Conducting a community readiness assessment is an important part of the program planning process as it allows intervention staff to tailor intervention strategies based on the community’s readiness to change. Involving community members in defining the issue and the parameters for community will help make intervention strategies locally relevant and thus improve community ownership and integration of the health promotion program. Externally imposed interventions risk wasting resources on strategies for which the community isn’t ready. The CRT can be used not only as a way to inform interventions but also as part of the evaluation to monitor change in readiness over time.

Although the CRT was designed to be adaptable to different issues and contexts, our review suggests that it has been applied in ways that deviate quite significantly from the specified protocol. More specifically, 40% of studies modified the CRT beyond what the protocol identifies as acceptable adaptation to the local issue and community. One study did not conduct any interviews and used the CRT as a narrative tool to describe the changes that occurred in a community [27]. Another study only assessed leadership, conducted a single interview for each community and then assigned a community readiness score between 0 and 1 [23]. It is noteworthy to point out that some studies changed the CRT either by adding new dimensions or changing the wording of existing dimensions. Jason *et al.* separated the Climate dimension into Town Climate and Police Department climate to reflect the differences between those two sections of the community [15]. York et al did not use the Knowledge of Existing Efforts dimension, and added a new Political Climate dimension [32]. Interestingly, these applications were focused on the passage and/or implementation of tobacco policies; thus, researchers may have felt that modifications or additions to the climate dimension were necessary for a comprehensive readiness assessment. These applications point to a potential for future useful expansion of the model related to policy readiness.

Methodological modifications included changing interviews from individual to group interviews, conducting a single interview, using an online questionnaire which then computed readiness scores, and/or replacing or removing core questions. Whilst there is evidence to suggest that changing administration methods yields similar or even improved performance [33], most changes to the CRT were done without validating the new protocols. Major changes to the protocol, such as altering or removing core questions, utilising untested administration methods, or modifying dimension definitions call into question the validity of results. The changes made may reflect the time and resource intensity of the CRT data collection process and the subsequent delay in giving feedback to the community. Given that a frequently cited benefit of the tool was its ability to offer locally tailored intervention strategies, prompt completion of the CRT and return of information to the community is of upmost importance. These results suggest that future modifications to the CRT are required to improve its fit for purpose or its on-the-ground financial, resource and time efficiencies.

Even among studies which did not make significant changes to the methodology, the reporting of community readiness scores was inconsistent and often unclear. Some studies provided full tables of dimension scores for each community, whilst others reported only overall scores. In some cases, results were reported only in text or graphical format. In addition, the standard deviations for the scores were not reported in most studies. This is an important limitation given the utility of the variability of scores.

As a starting point for improving the quality reporting of CRT studies, we propose the set of recommendations outlined in Table 3. These recommendations were modelled after existing reporting guidelines, such as the CONSORT [34] and PRISMA [35] statements for randomised controlled trials and systematic reviews, respectively. Clear definitions of the communities and the issues are requisite for contextualising use of the CRT. Information on sampling key informants, the interview process and methodology will allow for better replication of studies, as well as highlight any changes which could impact on the transferability of findings. Reporting of overall scores and dimension scores and standard deviations in table form will enable meta-analysis of data as well remove any ambiguity from text or graphical only reporting.

To understand the nature of change in community readiness over time, this review aimed to synthesise the results of studies applying the CRT at multiple time points. Our review identified six studies applying the CRT in this way [5,15,19,20,21,22]. Unfortunately, it was not possible to synthesise results across studies due to reporting limitations. Having the information on community readiness before and after an intervention provides insight into the intervention duration required for change to happen in intervention communities compared to control/comparison communities, and the types of strategies and resourcing required to mobilise communities.

**Table 3:** Reporting recommendations for primary studies which apply the community readiness tool (CRT).

Section	Descriptor
*Title and Abstract*	
	Identify use of the CRT in the title of the paper
	Identify community
	Identify issue
*Introduction*	
Background	Provide rationale for application of the CRT and CRM in relation to the issue
*Methods*	
Context of Application	The community is clearly define
	The readiness issue is clearly defined
Objectives	Specific objectives and hypotheses pertaining to the CRT are provided
Participants	Key respondents’ eligibility criteria are clearly outlined
	The recruitment method including a sampling method is provided
Data Collection	A statement of interviewer qualifications is provided
	The number of interviews for each community is stated
	Any modifications to the core questions, protocol or dimensions outlined in the CRT handbook is outlined with justifications provided for each change
Scoring	The qualifications of the scorers is provided
	Any deviations to the scoring protocol is outlined and justified
*Results*	
Participants	Participant response rate is reported
Data	Numerical representation of the overall community readiness score (mean and standard deviation) and each dimension score is reported in table form
	The corresponding readiness stages to the overall and dimension scores are clearly presented
*Discussion*	
Interpretation	Results are interpreted in relation to study objectives and hypotheses
	Results are interpreted with consideration to changes to the tool
	Discussion of research, practice and policy implications
Generalizability	Discussion of the generalisability of the results, taking into account the community and issue, length of follow-up and other contextual issues
Overall Evidence	The CRT and CRM results are interpreted in the context of existing CRT and CRM applications and broader evidence on the topic

The six evaluation studies which assessed community readiness at multiple time points consistently praised the CRT for its ability to “*kick-start*” prevention efforts by identifying key stakeholders, engaging the community, and informing intervention staff of the types of projects which are likely to succeed. The mere act of conducting a community readiness assessment helped improve awareness of the issue in leaders and stakeholders. Slater *et al.* [5] suggests that the longer a community is exposed to a program, the greater the increase in community readiness, with an increase of approximately 0.6 community readiness levels per year. This tends to hold for the other studies, with all reporting an increase of between 0.5 and 1 community readiness levels per year of intervention, with the exception of Allen *et al*. [20], who reported an increase of 2.1 following one intervention year. Although interventions can lead to increases in community readiness in a relatively short period of time, a longer time period appears to be required to observe changes in health outcomes (e.g., suicide, obesity, CVD rates). The results from Millar [19] and, to a lesser extent, Peercy [22] suggest that a positive change in health outcomes (childhood obesity and heart health respectively) is associated with a community readiness level of at least 5 (preparation). However, further research is needed to corroborate these results. This finding may be of direct relevance to public health and health promotion intervention planning, as it provides a threshold against which communities may expect to see positive changes in health outcomes.

The report by Slater *et al.* [5], highlights the statistical power issues faced when community is the unit of analysis. Large-scale intervention studies are costly, and a large number of communities are required to find the often small but meaningful changes in outcomes at the population level. Millar [19] experienced similar issues; the time and resource costs limited the number of communities which could be assessed and made it difficult to return feedback to communities in a timely manner. However, the number of communities in each study was consistently relatively small. Assessment in larger scale quasi-experimental designs is challenging in a climate where resources for evaluation are scarce.

The strengths of the CRT and CRM were recognised by the majority of studies, however limitations were discussed infrequently. Space limits in publications may have contributed to the infrequent reporting. Alternatively, those applying the tool may have been satisfied with the CRM and CRT or hesitant to question the methodology, anticipating that it may undermine the validity or merit of their application.

### 3.2. Limitations

There are two key limitations to the present review. First, the search strategy only included peer review published applications of the tool and did not consider applications in the grey literature (e.g., government and community reports). Second, the review is limited to the extent that the search strategy did not retrieve publications which used the CRT but did not mention it in the title, abstract or keywords. In terms of study strengths, searching a broad range of databases and the use of simple search terms (without restrictions) provides some assurance that the majority of published applications were captured. Having two independent reviewers extract information from all 40 studies is an additional study strength.

## 4. Conclusions

Although the readiness scores from the six studies applying the CRT over time could not be synthesised, the results are promising. Changes in readiness can be observed after as little as one year of intervention, with health and social outcomes following increases of readiness to the level of preparation (scores of 5 and above). Application of the tool on a large scale will always be time and resource intensive unless the tool is substantially revised. Changes to the CRT which reduce response burden, scoring time and logistical difficulties whilst maintaining methodological rigour and construct validity may facilitate its uptake in the planning and evaluation of public health intervention programs in single communities and larger scale studies where the community is the unit of analysis. In addition, the inclusion of dimensions that explicitly account for the political community climate may prove useful to those assessing readiness for policy changes and implementation.

## Supplementary Files

Supplementary File 1
