# Supplementary material for: A Systematic Review of Community Readiness Tool Applications: Implications for Reporting
Source: Int J Environ Res Public Health. 2015 Mar 24;12(4):3453–68. doi: 10.3390/ijerph120403453 (PMC4410196; doi:10.3390/ijerph120403453)
Supplement: Supplementary File 1 [file ijerph-12-03453-s001.pdf]

A Systematic Review of Community Readiness Tool Applications: Implications for Reporting

Table S1. Extraction of variables for year of publication, readiness issue, country, definition of community, location, impetus for usage, number of communities, interviews per community, format of reporting of results and range of overall community readiness scores.

Year and Reference	Readiness Issue	Country	Definition of Community	Location (Urban/ Rural)	Impetus for Usage	Number of Communities	Interviews per Community	Format of Reporting of Results	Range of overall Community Readiness Scores (Values Reported)
2010 [1]	HIV/Aids	Bangladesh	Administrative units	Rural	Planning of future prevention efforts	2	3 (6 total)	Results reported separately for each of the interviews. Modal and maximum scores presented on each dimension.	Scoring was different and makes for difficult comparisons.
2009 [2]	Suicide and alcohol abuse	USA	Rural towns	Rural	Pre- and Post-intervention readiness assessment	1	3–5 (8 total)	Average scores for each dimension pre and post intervention. Text only.	3.5–5.6
2012 [3]	Varied	USA	Health Centres	Urban	Planning of future prevention efforts	15	4–6 (79 total)	Mean, range and SD for each of the two types of communities. Means for each dimension.	2.6–7.5
2007 [4]	Breast health	USA	Ethnic group	Both	Planning of future prevention efforts	4	4–5 (19 total)	Stages of readiness only briefly discussed, no actual data.	2.0–7.0
2003 [5]	Intimate partner violence	USA	Administrative units	Urban	Planning of future prevention efforts	5	5–6 (total 26)	Bar graph of the different dimensions for the whole city, no data on separate community scores.	None reported.
2011 [6]	Provision of services GLBD	USA	Long term care facility	Urban	Planning of future prevention efforts	1	6	Each dimension score given in-text.	2.0
2013 [7]	Children’s physical activity	USA	Schools	Both	Program Evaluation	17	6–7 (101 total)	Overall CR score for each school given.	2.4–4.6

Table S1. *Cont.*

Year and Reference	Readiness Issue	Country	Definition of Community	Location (Urban/ Rural)	Impetus for Usage	Number of Communities	Interviews per Community	Format of Reporting of Results	Range of overall Community Readiness Scores (Values Reported)
2002 [8]	Youth access to tobacco	USA	Country Towns	Rural	Planning of future prevention efforts	11	Unclear	Range, mean and SD given.	1.6–5.9
2012 [9]	Smoke free policies	USA	Counties	Rural	Planning of future prevention efforts	22	1–2 (32 total)	No scores given.	None reported.
2011 [10]	Trauma-Informed care	USA	Health Centre	Urban	Planning of future prevention efforts	1	7	Table showing each dimension score.	6.0
2007 [11]	Childhood obesity	USA	Union county	Rural	Planning of future prevention efforts; Community engagement	1	15	Overall score only.	1.6
2012 [12]	Childhood obesity	USA	Ethnic group	Urban	Planning of future prevention efforts	2	8–10 (18 total)	Table showing each dimension for each community.	2.0–3
2013 [13]	Smoke free policies	USA	Counties	Rural	To compare CR to a specific measure of tobacco control	30	8 (231 total)	Mean, SD and range of overall CR scores in Table.	2.1–3.8
2003 [14]	Domestic violence	USA	Ethnic group	Urban	Interpretation of previous data around prevention efforts	1	NA	Guessing at what stage the community is.	2.0 or 3.0
2008 [15]	General health	USA	Ethnic group	Urban	Planning of future prevention efforts	1	Unclear	Overall CR score given for both residents and leaders.	4.0–5.0
2011 [16]	Tobacco control policies	Canada	Indigenous communities	Rural	Planning of future prevention efforts	6	Unclear	No scores reported.	None reported.

Table S1. *Cont.*

Year and Reference	Readiness Issue	Country	Definition of Community	Location (Urban/ Rural)	Impetus for Usage	Number of Communities	Interviews per Community	Format of Reporting of Results	Range of overall Community Readiness Scores (Values Reported)
2004 [17]	Youth tobacco access	USA	Rural towns	Rural	To evaluate CR as an indicator of program success.	11	Unclear	Only change in scores reported.	None reported.
2012 [18]	Legal drug use	USA	Rural towns	Rural	Planning of future prevention efforts	Unclear	8	No CR results presented.	None reported.
2008 [19]	Bicycle helmet use	USA	Shared interest group	Urban	Planning of future prevention efforts	10	4–25 (85 total)	Some overall scores given, some individual dimension scores given.	2.0–3.0
2004 [20]	HIV/Aids	Liberia	Urban city	Urban	Planning of future prevention efforts	1	12	Table with dimension and overall CR scores.	3.6
2009 [21]	Youth tobacco access	USA	Rural towns	Rural	To evaluate community context before intervention	24	1	Text only. Range, mean and SD given.	2.3–6.3
2007 [22]	Breast cancer research	USA	Ethnic group	Both	To inform researchers on the factors behind Latino women's participation in cancer clinical trials	4	5 (19 total)	Table of scores by community and dimension.	3.3–4.2
2001 [23]	Drug court program	USA	Courts	Both	Planning of future programs	12	1	Table with counties and the overall score given.	1.0–4.0
2007 [24]	HIV/Aids	USA	Islands	Rural	Planning of future prevention efforts	3	2–5 (12 total)	Table giving overall and dimension score for each community.	3.8–4.7
2011 [25]	Disability	India	Rural towns	Rural	Planning of future prevention efforts	3	10 (30 total)	Bar chart showing each of the dimension scores for each village.	2.5–3.5

Table S1. *Cont.*

Year and Reference	Readiness Issue	Country	Definition of Community	Location (Urban/ Rural)	Impetus for Usage	Number of Communities	Interviews per Community	Format of Reporting of Results	Range of overall Community Readiness Scores (Values Reported)
2013 [26]	Childhood obesity	Australia	Schools	Rural	Pre- and Post- intervention readiness assessment	12	4–5 (108 total)	Baseline and follow up overall scores. Intervention and comparison aggregate dimension scores.	2.0–6.0
2003 [27]	Native American cultural programs	USA	Ethnic group	Urban	Planning of future prevention efforts	1	15	Range of overall scores given in-text only.	6.0–9.0
2008 [28]	Use of harmful legal products by minors	USA	Rural towns	Rural	Planning of future prevention efforts	4	8 (32 pre and 34 post)	Table with pre and post readiness scores.	2.9–3.8
2011 [29]	Youth violence	USA	Urban city	Urban	As a theoretical model to understand the outcomes and events of a failed prevention program	1	0	Vague in-text scores talked about. (guessing of scores)	None reported.
2010 [30]	Prevention of CVD	USA	Ethnic group	Rural	Pre- and Post-intervention readiness assessment; Planning of prevention efforts	1	33 pre and 33 post	In-text, also a bar graph showing each individual dimension and overall scores.	4.6–5.8

Table S1. *Cont.*

Year and Reference	Readiness Issue	Country	Definition of Community	Location (Urban/ Rural)	Impetus for Usage	Number of Communities	Interviews per Community	Format of Reporting of Results	Range of overall Community Readiness Scores (Values Reported)
1999 [31]	Drug use	USA	Ethnic group	Rural	To evaluate differences between communities in respect to drug use programs; To inform the planning of future prevention efforts	102	3 (308 total)	Line graph showing proportion of scores at each of the 9 stages of readiness broken down by community type.	1.0–8.0
2007 [32]	HIV/Aids	USA	Rural towns	Rural	Planning of future prevention efforts	30	4 (120 total)	Table showing the number of communities in each stage of overall readiness, as well as each dimension stratified by community ethnicity.	2.0–5.0
2009 [33]	Cancer Health	Not clear	Communities experiencing cancer health disparities	Unknown	To advance the understanding of the protocols, consensus strategies and scoring challenges involved in determining the CR	5	6–10 (unclear total)	Not reported (paper focused on scoring process).	None reported.
2010 [34]	Cancer health	USA	Ethnic group	Both	Planning of future prevention efforts	1	8	Overall score given in-text.	3.0
2005 [35]	Substance abuse	USA	Not clear	unknown	Pre- and Post- intervention readiness assessment AND evolution of intervention strategies	16	6 (112 pre and 92 post)	Table showing scores for each of the dimension scores stratified by pre, post, intervention and control. Separate table stratifying dimension scores by informant affiliation.	None reported.

Table S1. *Cont.*

Year and Reference	Readiness Issue	Country	Definition of Community	Location (Urban/ Rural)	Impetus for Usage	Number of Communities	Interviews per Community	Format of Reporting of Results	Range of overall Community Readiness Scores (Values Reported)
2011 [36]	Childhood obesity prevention	USA	Urban city	Urban	To assess and select communities for intervention	10	4 (40 total)	In-text reporting of average scores and range of overall and dimension scores.	3.0–5.4
2008 [37]	Traumatic brain injury prevention	USA	Counties	Rural	Pre- and Post- intervention readiness assessment AND evaluation of intervention strategies	4	4–6 (21 pre and 19 post)	Table showing the level each community achieved pre and post intervention.	2.0–5.0
2012 [38]	Intimate partner Violence	USA	Sexual orientation	Both	Planning of future prevention efforts, specifically which community to intervene on	16	5 (81 total)	Table showing scores for each of the 16 communities.	2.0–4.0
2010 [39]	Alcohol related harm	Australia	Country town	Rural	Planning of future prevention efforts	1	16	Table showing scores for each dimension and overall.	3.0
2008 [40]	Smoke free policies	USA	Counties	Both	To evaluate smoke free policy development at the local level	64	1	Bar graphs and tables on the dimension and overall scores.	Scoring completely changed.

Table S2. Extraction of variables for year of publication, modification of the tool, reason for modification, Modification description, perceived strengths and limitations of the Community Readiness Tool (CRT) and Community Readiness Model (CRM).

Year and Reference	Modification of tool (Y/N)	Reason for modification	Modification Description	Perceived strengths of CRT	Perceived limitations CRT	Perceived strengths CRM	Perceived limitations CRM
2010 [1]	Y	Adapt to local context and improve respondent participation.	Group interview in lieu of individual interviews. Scoring process changed from a score out of 9 to a score out of 4. Dimensions were changed.	Allows for assessment of the community's willingness and ability to support programs.	Methods need to be changed to obtain necessary information.	None reported.	None reported.
2009 [2]	N	NA	NA	None reported.	None reported.	None reported.	None reported.
2012 [3]	N	NA	NA	Engages the community and builds partnerships. Validated measure with multiple respondents. Feedback to the community.	None reported.	None reported.	None reported.
2007 [4]	N	NA	NA	None reported.	None reported.	Wide range of sources of information. Previous success. Avoids the imposition of worldviews.	None reported.
2003 [5]	N	NA	NA	None reported.	None reported.	Viable way to assess and develop interventions with diverse groups across cultures.	None reported.
2011 [6]	N	NA	NA	The tool helped the facility to critically examine their practices and culture.	Time commitment is high.	Objective and reliable results from a small sample.	None reported.
2013 [7]	Y	Minimising time constraints.	Modification of core questions after a pilot study.	None reported.	High time and resource commitment. Calls for shorter CR tool.	Theory-based method to understand readiness to change.	None reported.

Table S2. *Cont.*

Year and Reference	Modification of tool (Y/N)	Reason for modification	Modification Description	Perceived strengths of CRT	Perceived limitations CRT	Perceived strengths CRM	Perceived limitations CRM
2002 [8]	Y	To improve the fit with the specific topic.	Community climate split into two different dimensions - town climate and police department climate.	None reported.	None reported.	Unique contextual factor. Has roots in community development. Provides tailored intervention strategies.	None reported.
2012 [9]	Y	Make the existing tobacco CRT easier/shorter.	Instead of the usual phone interview, 61 online questions were asked.	Long version is not online (more accessible) and gives quantitative data.	Long version is time and resource intensive.	None reported.	None reported.
2011 [10]	N	NA	NA	Culturally sensitive and strength based tool.	None reported.	Provides a guided assessment and intervention process based on current level of readiness.	None reported.
2007 [11]	Y	Better suit local context.	Made changes to core questions.	Processes helped find coalition members and interest for initiating childhood obesity prevention. Easily adaptable. Provided rich data to be analysed qualitatively. Scoring easy to conduct.	None reported.	None reported.	None reported.

Table S2. *Cont.*

Year and Reference	Modification of tool (Y/N)	Reason for modification	Modification Description	Perceived strengths of CRT	Perceived limitations CRT	Perceived strengths CRM	Perceived limitations CRM
2012 [12]	N	NA	NA	Provides a framework for matching interventions to the community. Effective way of ensuring community ownership. Provides a useful measure of social change.	None reported.	Informs future programs and strategies.	None reported.
2013 [13]	N	None given.	Dimensions changed. Each score then rescaled between 0 and 1.	None reported.	Not previously used to study public policy development. Response bias.	Provides a theoretical basis for understanding policy change. Builds on research traditions.	None reported.
2003 [14]	N	Data availability.	CR given a score from other data already collected, no interviews or formal scoring process.	None reported.	None reported.	Creating a shared vision for the community. Quickly taken up by many fields.	Model is young.
2008 [15]	N	NA	NA	None reported.	None reported.	Model is adaptable to new communities.	None reported.
2011 [16]	N	NA	NA	None reported.	None reported.	Provides starting point in understanding of the process involved in changing tobacco control policies.	Not comprehensive enough. Economic and social factors are not clearly reflected in the model.

Table S2. *Cont.*

Year and Reference	Modification of tool (Y/N)	Reason for modification	Modification Description	Perceived strengths of CRT	Perceived limitations CRT	Perceived strengths CRM	Perceived limitations CRM
2004 [17]	N	To improve the fit with the specific topic.	Community climate split into two different dimensions-town climate and police department climate.	None reported.	None reported.	None reported.	None reported.
2012 [18]	N	NA	NA	None reported.	None reported.	None reported.	None reported.
2008 [19]	N	NA	NA	None reported.	None reported.	Allows researchers to understand the community context in which programs are implemented.	None reported.
2004 [20]	N	Adaptation to new context.	Paper based self-administered survey rather than interview.	None reported.	None reported.	Identifies specific characteristics regarding different levels of problem awareness and readiness to change.	None reported.
2009 [21]	Y	To improve the fit with the specific topic.	Separation of town climate and police department climate. Questions changed to yes no to speed up scoring process.	Rich qualitative results. Reliable and valid measure of readiness to implement important public health initiatives.	Resource intensive task for completing and scoring interviews.	None reported.	None reported.

Table S2. *Cont.*

Year and Reference	Modification of tool (Y/N)	Reason for modification	Modification Description	Perceived strengths of CRT	Perceived limitations CRT	Perceived strengths CRM	Perceived limitations CRM
2007 [22]	N	NA	NA	Effective theoretical and data collection method, helped formulate suggestions regarding possible interventions.	None reported.	None reported.	Limited to the 6 dimensions, information may be missed. Reliance on key informants.
2001 [23]	Y	Not stated.	Different administration method-15 min interview with each judge used to obtain a rating on the 9 point CR scale.	None reported.	None reported.	None reported.	None reported.
2007 [24]	N	NA	NA	Identifies resources. Facilitates community ownership, no outside experts necessary.	None reported.	Provides tailored, sustainable and develops culturally appropriate strategies intervention strategies. Useful for community evaluation research.	None reported.
2011 [25]	Y	Different culture.	Translated and adapted to local culture.	Facilitates community engagement; Provides a starting place for intervention planning; Dimension scores give rich community data.	Some questions not understood.	None reported.	None reported.

Table S2. *Cont.*

Year and Reference	Modification of tool (Y/N)	Reason for modification	Modification Description	Perceived strengths of CRT	Perceived limitations CRT	Perceived strengths CRM	Perceived limitations CRM
2013 [26]	N	NA	NA	Widely used and flexible for issues; Informative and reliable.	Time consuming to implement and score. Subjective Scoring. Dimensions don't cover all aspects of community capacity building.	None reported.	None reported.
2003 [27]	N	NA	NA	None reported.	None reported.	Innovative methodology, adapted to local context.	None reported.
2008 [28]	N	NA	NA	None reported.	None reported.	Flexibility of the model. Good starting point and goal for programs. Results engaged the community.	Development not rigorous, selection of key informants inconsistent. Singling out issues that might go together. Needs more validation.
2011 [29]	Y	Not stated.	No interviews, just using information to guess the score.	None reported.	None reported.	Useful for understanding the series of events that occurred during this prevention program.	None reported.
2010 [30]	N	NA	NA	None reported.	None reported.	Giving the "*truth of the community*" which is important when seeking community involvement.	None reported.

Table S2. *Cont.*

Year and Reference	Modification of tool (Y/N)	Reason for modification	Modification Description	Perceived strengths of CRT	Perceived limitations CRT	Perceived strengths CRM	Perceived limitations CRM
1999 [31]	N	NA	NA	None reported.	None reported.	Provides information to set up prevention efforts and to understand community dynamics.	None reported.
2007 [32]	N	NA	NA	Process used to teach the principals of the theory to local leaders and help them move stepwise through the stages of readiness. The method is economical and supports local control.	None reported.	Provides important information needed for developing prevention efforts.	None reported.
2009 [33]	N	NA	NA	Multiple scorers help overcome subjectivity. Qualitative instrument which uses leaders as key informants.	Subjective scoring; sometimes there is insufficient information to score.	None reported.	None reported.
2010 [34]	Y	Some questions inappropriate for the community.	9 questions removed, 4 added (unknown which ones).	Provides a wealth of other qualitative data.	None reported.	Matching of interventions to stage of readiness likely to lead to success.	None reported.

Table S2. *Cont.*

Year and Reference	Modification of tool (Y/N)	Reason for modification	Modification Description	Perceived strengths of CRT	Perceived limitations CRT	Perceived strengths CRM	Perceived limitations CRM
2005 [35]	N	NA	NA	Provides rich data. Rigorous coding procedures.	Power issues resulting from low number of interviews/ communities.	Provides a comprehensive framework that takes into account both dimensions and stages.	None reported.
2011 [36]	Y	To improve the fit with the specific topic.	Replaced 1 core question with another.	Consensus process overcomes subjective scoring.	Subjective scoring. Captures community at a single point, when CR is in constantly changing.	Valuable information on the community is gathered. Provides information that makes for a more efficient use of resources.	None reported.
2008 [37]	N	NA	NA	Sensitive to short term changes.	None reported.	Provides tailored intervention suggestions. Good for assessing community awareness and resource availability.	None reported.

Table S2. *Cont.*

Year and Reference	Modification of tool (Y/N)	Reason for modification	Modification Description	Perceived strengths of CRT	Perceived limitations CRT	Perceived strengths CRM	Perceived limitations CRM
2012 [38]	N	NA	NA	None reported.	Scheduling challenges, definition of community remained broad.	Assess each community/group separately. Engages community members, tailored intervention strategy based on CR score.	None reported.
2010 [39]	N	NA	NA	None reported.	None reported.	Evidence based tool used to help inform prevention strategies.	None reported.
2008 [40]	Y	To improve the fit with the specific topic.	Questions and dimensions revised and changed. Only one key informant per community used.	None reported.	None reported.	None reported.	Narrow definitions of stages of readiness-limiting accuracy of assessment.

References

1. Aboud, F.; Huq, N.L.; Larson, C.P.; Ottisova, L. An assessment of community readiness for HIV/AIDS preventive interventions in rural Bangladesh. *Soc. Sci. Med.* **2010**, *70*, 360–367.
2. Allen, J.; Mohatt, G.; Fok, C.C.T.; Henry, D.; People Awakening Team. Suicide prevention as a community development process: Understanding circumpolar youth suicide prevention through community level outcomes. *Int. J. Circumpol. Health* **2009**, *68*, 274–291.
3. Battaglia, T.A.; Murrell, S.S.; Bhosrekar, S.G.; Caron, S.E.; Bowen, D.J.; Smith, E.; Kalish, R.; Rorie, J.-A. Connecting Boston's public housing developments to community health centers: Who's ready for change? *Prog. Community Health Partnersh.* **2012**, *6*, 239–248.
4. Borrayo, E.A. Using a community readiness model to help overcome breast health disparities among U.S. Latinas. *Subst. Use Misuse* **2007**, *42*, 603–619.
5. Ogilvie, K.A.; Moore, R.S.; Ogilvie, D.C.; Johnson, K.W.; Collins, D.A.; Shamblen, S.R. Changing community readiness to prevent the abuse of inhalants and other harmful legal products in Alaska. *J. Community Health* **2008**, *33*, 248–258.
6. Carlson, L.A.; Harper, K.S. One facility's experience using the community readiness model to guide services for gay, lesbian, bisexual, and transgender older adults. *ADULTSPAN J.* **2011**, *10*, 66–77.
7. Ehlers, D.K.; Huberty, J.L.; Beseler, C.L. Is school community readiness related to physical activity before and after the ready for recess intervention? *Health Educ. Res.* **2013**, *28*, 192–204.
8. Engstrom, M.; Jason, L.A.; Townsend, S.M.; Pokorny, S.B.; Curie, C.J. Community readiness for prevention: Applying stage theory to multi-community interventions. *J. Prevent. Interv. Community* **2002**, *24*, 29–46.
9. Fallin, A.; Zuercher, R.; Rayens, M.K.; Adkins, S.; York, N.; Hahn, E.J. A short online community readiness survey for smoke-free policy. *Nicotine Tob. Res.* **2012**, *14*, 1494–1498.
10. Farro, S.A.; Clark, C.; Eyles, C.H. Assessing trauma-informed care readiness in behavioral health: An organizational case study. *J. Dual Diagn.* **2011**, *7*, 228–241.
11. Findholt, N. Application of the community readiness model for childhood obesity prevention. *Public Health Nurs.* **2007**, *24*, 565–570.
12. Frerichs, L.; Brittin, J.; Stewart, C.; Robbins, R.; Riggs, C.; Mayberger, S.; Cervantes, A.; Huang, T. SaludableOmaha: Development of a youth advocacy initiative to increase community readiness for obesity prevention, 2011–2012. *Prev. Chronic Dis.* **2012**, *9*, doi:10.5888/pcd9.120095.
13. Hahn, E.J.; Rayens, M.K.; York, N. Readiness for smoke-free policy and overall strength of tobacco control in rural tobacco-growing communities. *Health Promot. Prac.* **2013**, *14*, 238–246.
14. Han, M. Community readiness: A promising tool for domestic violence prevention programs in the Korean community. *J. Community Prac.* **2003**, *11*, 55–69.
15. Hull PC, Canedo J, Aquilera J, Garcia E, Lira I, Reyes F. Assessing community readiness for change in the Nashville Hispanic community through participatory research. *Prog. Community Health Partnersh.* **2008**, 185–194.
16. Hutchinson, J.; Bottorff, J.; Chambers, M.; Mowatt, R.; Wardman, D.; Sullivan, D.; Williams, W. What are the odds? Community readiness for smoke-free bingos in first nation communities. *Int. J. Indigenous Health* **2011**, *7*, 32–41.

17. Jason, L.A.; Pokorny, S.B.; Kunz, C.; Adams, M. Maintenance of community change: Enforcing youth access to tobacco laws. *J. Drug Educ.* **2004**, *34*, 105–119.
18. Johnson, K.W.; Grube, J.W.; Ogilvie, K.A.; Collins, D.; Courser, M.; Dirks, L.G.; Ogilvied, D.; Driscoll, D. A community prevention model to prevent children from inhaling and ingesting harmful legal products. *Eval. Program Plan.* **2012**, *35*, 113–123.
19. Kakefuda, I.; Stallones, L.; Gibbs, J. Readiness for community-based bicycle helmet use programs: A study using community-and individual-level readiness models. *J. Health Psychol.* **2008**, *13*, 639–643.
20. Kennedy, S.B.; Johnson, K.; Harris, A.O.; Lincoln, A.; Neace, W.; Collins, D. Evaluation of HIV/AIDS prevention resources in Liberia: Strategy and implications. *AIDS Patient Care Stds* **2004**, *18*, 169–180.
21. Kunz, C.B.; Jason, L.A.; Adams, M.; Pokorny, S.B. Assessing police community readiness to work on youth access and possession of tobacco. *J. Drug Educ.* **2009**, *39*, 321–337.
22. Lawsin, C.R.; Borrayo, E.A.; Edwards, R.; Belloso, C. Community readiness to promote Latinas' participation in breast cancer prevention clinical trials. *Health Soc. Care Community* **2007**, *15*, 369–378.
23. Logan, T.K.; Williams, K.; Leukefeld, C. A statewide drug court needs assessment: Identifying target counties, assessing readiness. *J. Offender Rehabil.* **2001**, *33*, 1–25.
24. McCoy, H.V.; Malow, R.; Edwards, R.W.; Thurland, A.; Rosenberg, R. A strategy for improving community effectiveness of HIV/AIDS intervention design: The community readiness model in the Caribbean. *Substance Subst. Use Misuse* **2007**, *42*, 1579–1592.
25. McElroy, T.A.; Davis, A.; Hunt, C.; Dadul, J.; Stanba, T.; Larson, C. Navigating a way forward: Using focused ethnography and Community Readiness to study disability issues in Ladakh, India. *Disabil. Rehabil.* **2011**, *33*, 17–27.
26. Millar, L.; Robertson, N.; Allender, S.; Nichols, M.; Bennett, C.; Swinburn, B. Increasing community capacity and decreasing prevalence of overweight and obesity in a community based intervention among Australian adolescents. *Prev. Med.* **2013**, *56*, 379–384.
27. Nebelkopf, E.; King, J. A holistic system of care for Native Americans in an urban environment. *J. Psychoact. Drugs* **2003**, *35*, 43–52.
28. Ogilvie, K.A.; Moore, R.S.; Ogilvie, D.C.; Johnson, K.W.; Collins, D.A.; Shamblen, S.R. Changing community readiness to prevent the abuse of inhalants and other harmful legal products in Alaska. *J. Community Health* **2008**, *33*, 248–258.
29. Parker, R.N.; Alcaraz, R.; Payne, P.R. Community readiness for change and youth violence prevention: A tale of two cities. *Am. J. Community Psychol.* **2011**, *48*, 97–105.
30. Peercy, M.; Gray, J.; Thurman, P.J.; Plested, B. Community readiness: An effective model for tribal engagement in prevention of cardiovascular disease. *Fam. Community Health* **2010**, *33*, 238–247.
31. Plested, B.; Smithman, D.M.; Jumper-Thurman, P.; Oetting, E.R.; Edwards, R.W. Readiness for drug use prevention in rural minority communities. *Subst. Use Misuse* **1999**, *34*, 521–544.
32. Plested, B.A.; Edwards, R.W.; Thurman, P.J. Disparities in Community Readiness for HIV/AIDS prevention. *Subst. Use Misuse* **2007**, *42*, 729–739.
33. Schroepfer, T.A.; Sanchez, G.V.; Lee, K.J.; Matloub, J.; Waltz, A.; Kavanaugh, M. Community readiness assessment: The scoring process revisited. *J. Community Pract.* **2009**, *17*, 269–290.

34. Schroepfer, T.A.; Waltz, A.; Noh, H.; Matloub, J.; Kue, V. Seeking to bridge two cultures: The Wisconsin Hmong cancer experience. *J. Cancer Educ.* **2010**, *25*, 609–616.
35. Slater, M.D.; Edwards, R.W.; Plested, B.A.; Thurman, P.J.; Kelly, K.J.; Comello, M.L.; Keefe, T.J. Using community readiness key informant assessments in a randomized group prevention trial: Impact of a participatory community-media intervention. *J. Community Health* **2005**, *30*, 39–53.
36. Sliwa, S.; Goldberg, J.P.; Clark, V.; Collins, J.; Edwards, R.; Hyatt, R.R.; Junot, B.; Nahar, E.; Nelson, M.E.; Tovar, A. Using the Community Readiness Model to select communities for a community-wide obesity prevention intervention. *Prev. Chronic Dis.* **2011**, *8*, 1–9.
37. Stallones, L.; Gibbs-Long, J.; Gabella, B.; Kakefuda, I. Community readiness and prevention of traumatic brain injury. *Brain Injury* **2008**, *22*, 555–564.
38. Turell, S.; Herrmann, M.; Hollander, G.; Galletly, C. Lesbian, gay, bisexual, and transgender communities' readiness for intimate partner violence prevention. *J Gay Lesbian Soc. Serv.* **2012**, *24*, 289–310.
39. Velander, F.; Schineanu, A.; Midford, R. Attitudes and knowledge of alcohol related harm minimization initiatives in a rural Australian city: A baseline survey. *Drugs Educ. Prev. Policy* **2010**, *17*, 354–369.
40. York, N.L.; Hahn, E.J.; Rayens, M.K.; Talbert, J. Community readiness for local smoke-free policy change. *Am. J. Health Promot.* **2008**, *23*, 112–120.

© 2015 by the authors; licensee MDPI, Basel, Switzerland. This article is an open access article distributed under the terms and conditions of the Creative Commons Attribution license (http://creativecommons.org/licenses/by/4.0/).
